# Leverage zones in Responsible AI: towards a systems thinking conceptualization

**DOI:** 10.1057/s41599-023-01579-0

**Published:** 2023-03-04

**Authors:** Ehsan Nabavi, Chris Browne

**Affiliations:** Responsible Innovation Lab, Center for Public Awareness of Sciences, The Australian National University, Canberra, ACT Australia

**Keywords:** Science, technology and society, Business and management, Information systems and information technology

## Abstract

There is a growing debate amongst academics and practitioners on whether interventions made, thus far, towards Responsible AI have been enough to engage with the root causes of AI problems. Failure to effect meaningful changes in this system could see these initiatives not reach their potential and lead to the concept becoming another buzzword for companies to use in their marketing campaigns. Systems thinking is often touted as a methodology to manage and effect change; however, there is little practical advice available for decision-makers to include systems thinking insights to work towards Responsible AI. Using the notion of ‘leverage zones’ adapted from the systems thinking literature, we suggest a novel approach to plan for and experiment with potential initiatives and interventions. This paper presents a conceptual framework called the Five Ps to help practitioners construct and identify holistic interventions that may work towards Responsible AI, from lower-order interventions such as short-term fixes, tweaking algorithms and updating parameters, through to higher-order interventions such as redefining the system’s foundational structures that govern those parameters, or challenging the underlying purpose upon which those structures are built and developed in the first place. Finally, we reflect on the framework as a scaffold for transdisciplinary question-asking to improve outcomes towards Responsible AI.

## Introduction

The general public is becoming increasingly aware of how ingrained Artificial Intelligence (AI) already is in their daily lives—whether it determines what appears in a playlist or suggests potential partners to date—rather than in some distant future. While these seemingly low-risk examples can feel like magic to the user, many more technological advances are also underway that delegate more significant control over decision-making to AI-systems, such as in driving (Nunes et al., 2018), educating (Zawacki-Richter et al., 2019), judicial applications (Cui, 2020), and providing health care (Schwalbe & Wahl, 2020). Research into implications of AI can be seen in fields as diverse as health (Gupta et al., 2021; Trocin et al., 2021), finance (Maree et al., 2020), urban studies (Yigitcanlar et al., 2021), conservation science (Wearn et al., 2019), marketing (Liu et al., 2021), and military affairs (Stanley-Lockman & Trabucco, 2022), to more specific cases such as COVID-19 (Leslie, 2020).

However, it is increasingly well understood that AI applications can inadvertently erode the shared values of society, such as fairness, justice, safety, security, and accountability, and exacerbate other societal problems, such as loss of privacy through increased surveillance (Mitchell & Diamond, 2018), and policy decisions that increase social and economic inequality (Caetano & Simpson-Young, 2021; Perc et al., 2019; Walsh, 2020). Recent examples of AI failures and their lack of transparency and traceability have raised disconcerting questions about the ‘dark side’ of AI use (Mikalef et al., 2022), and the way these systems are developed and deployed (Choi, 2021).

Advances in digital technology, along with debates about biased algorithms and ethical and regulatory challenges of autonomous systems (Baker & Hawn, 2022; Coeckelbergh, 2019) underscore the fact that management of AI is as much a social and political issue rather than exclusively an engineering challenge (Coeckelbergh, 2022; Nabavi, 2019). This realization has caused policy, research, and industry actors to take non-technical aspects of AI into account, which can be seen in the increased awareness of ‘responsibility’ in AI systems (Constantinescu et al., 2021), defined broadly as including principles such as transparency, fairness, and accountability in creating AI technologies that meet legal requirements and societal expectation, norms and values. Common concerns in this area include privacy, accountability, safety and security, transparency and explainability, fairness and non-discrimination, human control of technology, professional responsibility, and promotion of human values (Fjeld et al., 2020).

Numerous frameworks, principles, guidelines and tools have been released by governments and leading organizations to address the ethical implications of AI-enabled systems (Hagendorff, 2020; Schiff et al., 2021). Table 1 highlights examples of such initiatives in relevant sectors. The nature, scope and locus of influence of these initiatives vary widely: from product level improvements emphasizing isolated factors supporting or hindering its implementation, such as bias, safety, privacy, and security (e.g., Gunning et al., 2019; Merhi, 2022; Rakova et al., 2021), approaches to transforming Responsible AI, such as enhancing principles and guidelines (IEEE, 2019; Jobin et al., 2019) and broader impacts of responsible digital footprints, such as those raised in corporate ecosystem responsibility (e.g., Wirtz et al., 2022) and digital sustainability (e.g., CODES, 2022).

**Table 1:** Measures and initiatives developed by different actor/sectors to create positive change in AI management, achieving more responsible outcomes.

Sector/description	Examples of existing initiatives
**Government:** Several governments have established the essential principles that underpin Responsible AI (Jobin et al., 2019). Scientific research organizations also helping the national government to develop operationalized guidelines for Responsible AI. OECD AI Policy Observatory reports there are more than 300 AI policy initiatives around the globe in this landscape (Ibaraki, 2021).	• European Commission tasked an independent expert group, to develop an integrative framework for responsible and trustworthy AI (EU, 2019).
	• In Australia, the national science agency, CSIRO (2022), uses the government’s AI Ethics Principles to develop a Responsible AI Pattern Catalog for operationalizing responsible AI (from software engineering perspective).
**Industry:** Major AI companies have launched self-regulatory Responsible AI programs, through building tools and software to translate responsibility principles such as fairness, explainability, and accountability and use them across engineering groups and clients, as shown in (de Laat, 2021) ‘s list of software tools. The major industry actors tend to engage by developing tangible products to solve the problem (Häußermann & Lütge, 2021; Scantamburlo et al., 2020; Schiff et al., 2020; Vyhmeister et al., 2022).	• Microsoft (2021) and Google (2022) provide resources and recommended practices to build fairness, interpretability, privacy, and security into AI systems.
	• Fairness tools: Google (Facets, What-if-tool (2021), Fairness Indicators); Microsoft (FairLearn); Facebook (Fairness Flow); IBM (AI Fairness 360 Toolkit); Salesforce (Einstein discovery tools).
	• Explainability tools: Amazon (SHAP); Microsft (InterpretML); IBM (AI Explainability 360 Toolkit); FaceBook (Captum); McKensy (CausalNex)
	• Accountability tools: Google (Model cards); Microsoft (Data sheets); IBM (Fact sheets).
**Academia:** In research, the notion of Responsible AI has attracted interest from fields as diverse as health (Gupta et al., 2021; Trocin et al., 2021), finance (Maree et al., 2020), urban studies (Yigitcanlar et al., 2021), conservation science (Wearn et al., 2019), marketing (Liu et al., 2021), and military affairs (Stanley-Lockman & Trabucco), to more specific cases such as COVID-19 (Leslie, 2020).	• Postgraduate coursework on Responsible AI (e.g., University of Queensland, UC Santa Cruz, Texas A&M University).
	• Curriculum design project (e.g., London New College of Humanities, 3Ai Institute at the Australian National University).
	• Interdisciplinary Research Center (e.g., Carnegie Mellon Responsible AI initiative, Cambridge responsible AI research center, RAISE at the University of Washington).
**Professional communities and institutes** offer guidance by publishing standards to describe technical specifications and procedures to develop Responsible AI systems. Certification processes is another movement to enhance assurance. Independent institutions and a number of government agencies have established their own assurance mechanism to provide a seal of trust to the stakeholders involved (e.g., MDIA, 2019). Consideration of broader implications that responsible AI has on other systems, such approaches for managing risk as part of corporate digital responsibility (e.g., Herden et al., 2021; Wirtz et al., 2022)and phased approaches to enabling global environmental sustainability (e.g., CODES 2022).	• Working groups associated with ISO (2021) and IEEE (2020, 2021) have published guidelines; for example: IEEE both provides a visionary documents on ‘ethically aligned design’ to show ethics in action (IEEE, 2019), and also provides more detail technical guidance into components, workflows, protocol, and security requirements for machine learning in which a model is trained using encrypted data [IEEE 2830-2021].
	• Responsible AI Institute, based in the US, gives RAII certification to an AI system, which is designed, developed, and deployed in line with the OECD principles on creating AI systems (RAII, 2022).
	• The International Corporate Digital Responsibility manifesto (CDR, 2021). outlines seven principles for the practices and behaviors to help an organization be perceived as socially, economically and environmentally responsible.

Consider a series of initiatives on the topic of fairness. Examples such as discriminatory algorithms, which mislabel people as primates (Cohn, 2019; Mac, 2021), or the screening algorithms, which discriminate against women candidates, (Chumley, 2018) act as a signal to improve the underlying pattens of behavior of factors such as ‘*fairness’* in AI systems. In public-facing applications of AI, initiatives are often reactive, and media and social media play a key role in determining which features are highlighted and attract attention and resources. These reactionary feedback cycles provide a cue for stakeholders about the type of remediation needed to meet societal expectations.

In response, a range of activity at different levels has propagated: research on the development of computational tools to evaluate and minimize such unfairness (Holstein et al., 2019) and other exploratory tools to mitigate bias before model training (FairLearn, 2021; Google, 2022, IBM, 2022); more inclusive policies on automated decision systems, particularly those used to classify people for the purpose of assessing their employment, insurance eligibility, and various government services (MacCarthy, 2019), and; new regulatory initiatives addressing algorithmic fairness, mandating companies to assess their AI systems for risks of “unfair, biased, or discriminatory decisions.” (US Congress, 2019, p. 3).

As it is clear in the case of improving fairness, initiatives can both align to reinforce efforts and work at cross-purposes. For example, regulatory standards encourage practitioners to effectively develop and use tools that mitigate bias throughout the AI application lifecycle while individual initiatives can result in fragmented solutions and misplaced efforts. Further, opinion is divided in the research community on the motivation for developing fairness tools, some describing them as ‘ethical washing’ or ‘ethics theatre’ (Bietti, 2020; Book, 2020; Mittelstadt, 2019) intended to show their customers they are doing their best to behave ethically and minimize the potential for regulation, and others arguing that efforts from industry actors thus far are ‘good first steps’ towards Responsible AI (de Laat, 2021).

These initiatives and efforts, ranging from loosely coordinated to independent, with multiple stakeholders, systems and interfaces leads us to a discussion of how we best intervene in a complex socio-technical system, the capacity of each initiative to improve Responsible AI, and how the initiatives might interact and their possible cumulative effects. Hence, in addition to efforts that encourage a broader and more critical conceptualization of Responsible AI (Mikalef et al., 2022), a shift towards improving Responsible AI also requires an understanding of managing complexity within ever-changing systems.

The United Nations Coalition for Digital Environmental Sustainability (CODES) highlights Systems Thinking as a tool in a required in the first of three phases in a shift towards a sustainable planet in the digital age (CODES, 2022). Formal systems thinking has multiple definitions and draws on methodology that spans a broad array of disciplines, approaches and applications, with many lineages in the natural, physical, social and design sciences.

To explore the potential applications of using systems thinking methodologies to align efforts towards Responsible AI, we identify two key challenges for managing complexity: how to work across disciplinary-based paradigms to effect positive change, and how to take a holistic view of the problem and solution space.

The first challenge identifies that the discourse for addressing AI problems is predominantly anchored in a disciplinary perspective, such as those seen in computer science and engineering. Even within the field of Responsible AI, researchers and practitioners tend to approach the topic from a narrowly disciplinary perspective and develop solutions based on their own epistemological strategies. For example, the priority of software developers is often to address visible gaps and tangible problems with technical improvements, such as updating existing systems with new software libraries (e.g., Soklaski et al., 2022). This is particularly common in areas such as robustness, privacy, and fairness where technical fixes seem feasible and the principles are easier to be quantified (Greene et al., 2019; Hagendorff, 2020). Counterintuitively, these fixes might also distract developers from taking a broader and structural view of the problem, by not effectively engaging with the root causes and unintended consequences, or question underlying assumptions about the vision and the purpose of the AI system.

The second challenge for the practitioner and policymaker is that the current body of literature does not adequately provide practical guidance taking a whole of systems view. For example, how to effectively apply systems thinking methodologies to the problem of Responsible AI without the need for intensive formal training. Although there are growing studies on adopting frameworks such as Responsible Innovation in which inclusivity, reflexivity, responsiveness and anticipation are considered (e.g., Tzachor et al., 2022), the research that explicitly focuses on a systems thinking understanding of Responsible AI are scarce and scattered. The literature also lacks a conceptual framework, or theoretical foundation, that allows to conceptualize, identify and evaluate the ‘effectiveness’ of interventions for Responsible AI in a structured way.

In this paper, we aim to address this gap by drawing on the insights about systems thinking from system dynamics literature—a field concerned with understanding the complex and dynamic relationships in socio-technical systems (see Maani & Cavana, 2007; Morecroft, 2015; Sterman, 2000). We propose an adaptation of Meadow’s (1999) work on leverage points from the systems thinking literature into a conceptual framework we have called the Five Ps, and explore its potential as a practical analytical and planning tool to situate, manage and align initiatives towards Responsible AI.

## Leverage zones to realize change

Meadows identifies twelve leverage points that has been adapted into research and practical work in various disciplines concerning complex socio-technical systems, from food and energy systems (Dorninger et al., 2020), to environmental systems (Rosengren et al., 2020), and health systems (Ramsey et al., 2019). As a collection, the leverage points represent common places to intervene within a system to effect change from adjusting parameters (described as low leverage) to transcending paradigms (described as high leverage).

As with any conceptual framework, care must be taken to evaluate whether leverage points are an effective mechanism to consider effecting change in a system. There is a notable absence in the literature of longitudinal experiments that demonstrate that a policy intervention is improved or otherwise different after taking into consideration Meadow’s leverage points. However, in the real-world policy intervention space, it is inconceivable to run controlled experiments that account for all the possible variables in a complex system and be certain of the outcomes. Thus, the value of a conceptual systems thinking framework is to explore potential hypotheses amidst large uncertainties and to organize shared thinking around a problem. Although over two decades old, the continued use of this conceptual framework demonstrates that it can be useful scaffold to consider the dynamics of complex systems for better planning and formulating interventions (Bolton, 2022; Riechers et al., 2022).

**Fig. 1:**
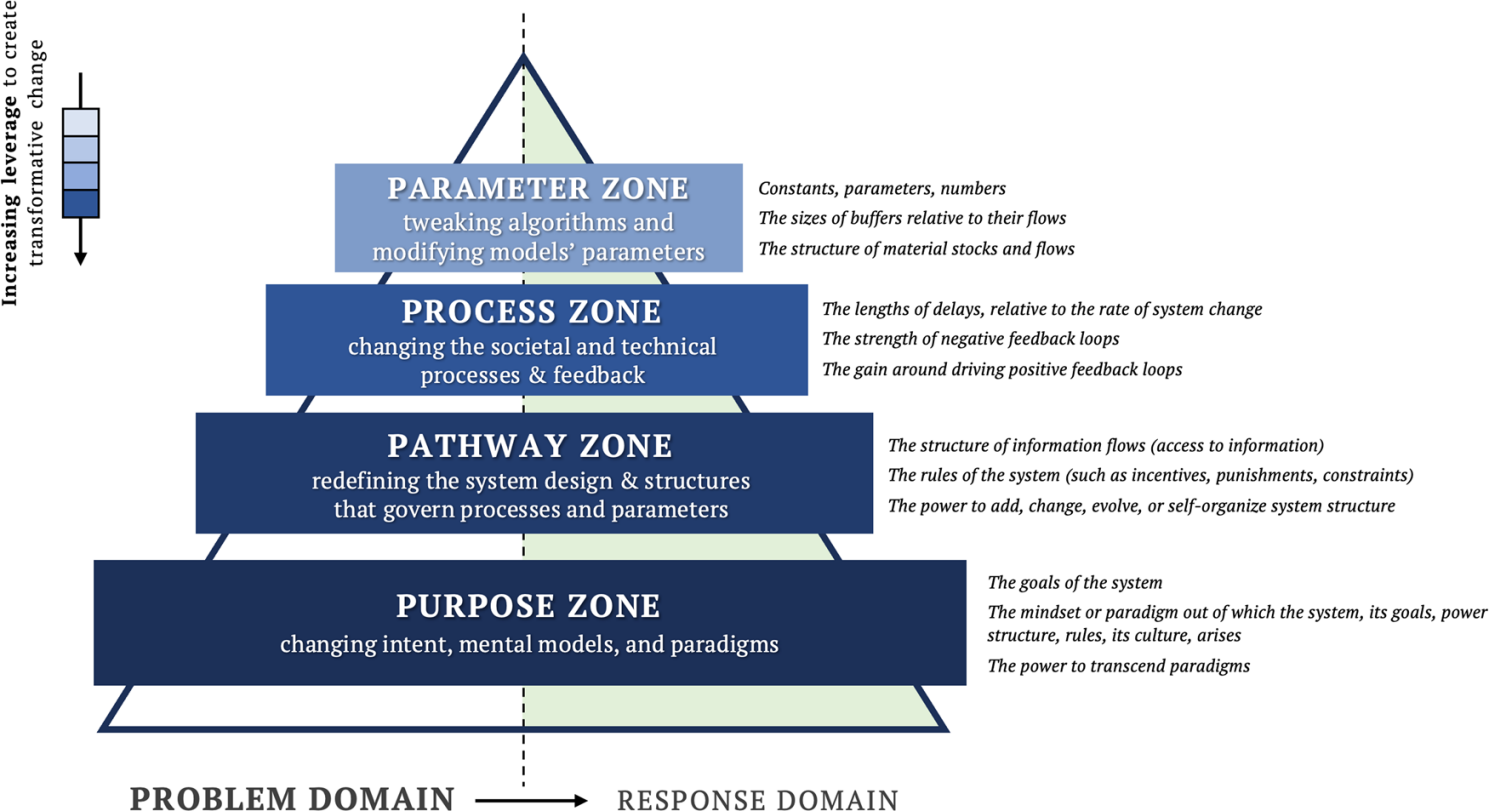
The 5Ps framework. The diagram illustrates the ‘leverage zones’ where interventions can be most effective. The lower zones in the pyramid offer greater leverage than the top zones. The leverage points corresponding to each leverage zone are displayed on the right side. According to Meadows (1999), these points indicate where interventions can be made in a system.

**Fig. 2:**
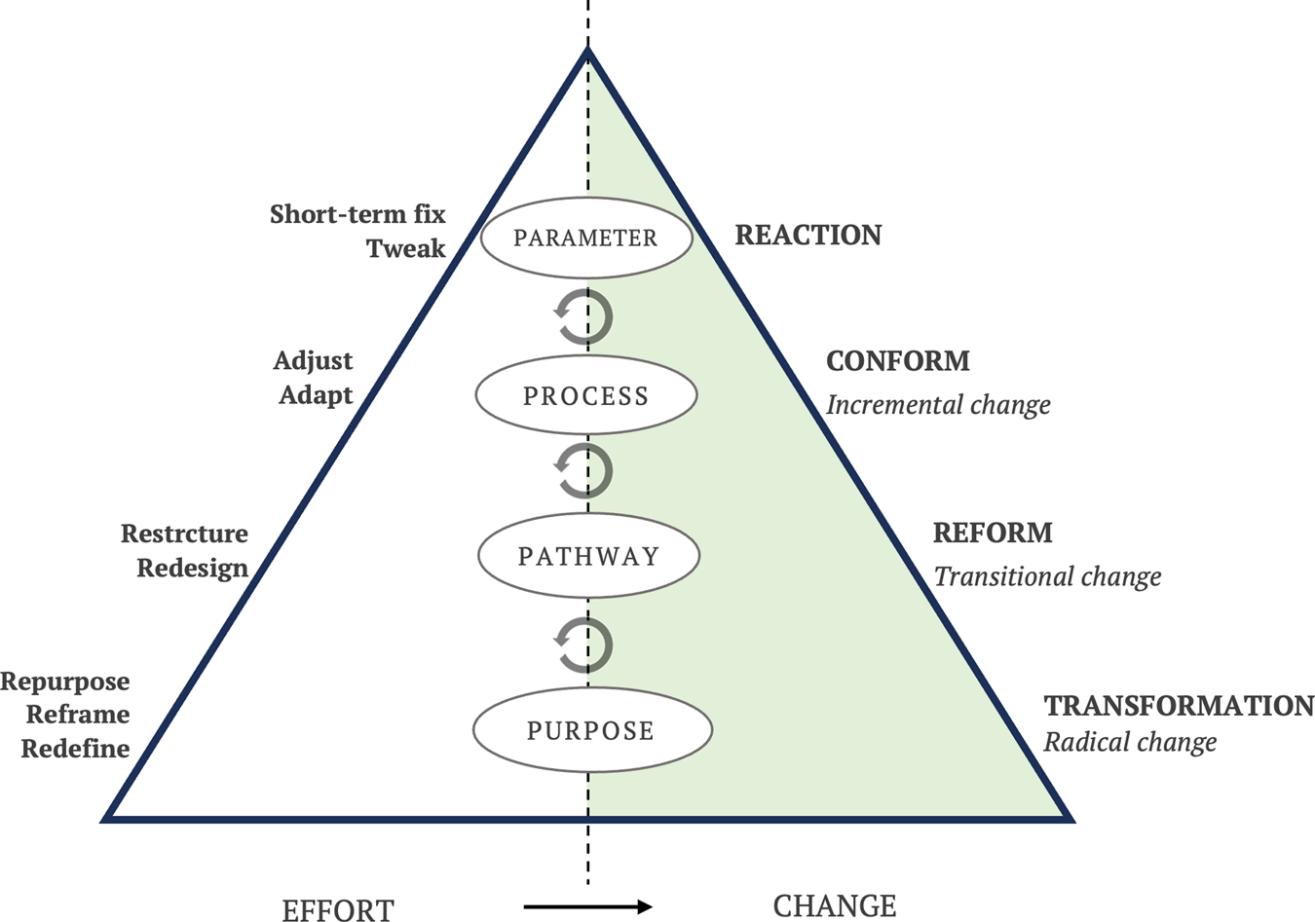
Leverage zones and their potential for different types of change. This is a schematic illustration of the leverage zones, showing their differences in terms of ‘efforts’ they are needed (on the left) and the type of ‘change’ they bring about (on the right). Feedback loops indicate interactions that may happen between and among different leverage zones.

In this paper we propose an adaptation of Meadow’s leverage point framework to help improve systems thinking literacy in relation to Responsible AI. Categorized around two domains and four zones that we call the Five Ps framework, depicted in Fig. 1. The two domains—Problem and Response—are represented by a triangle divided into two with the Problem Domain on the left and Response Domain on the right. The horizontal axis represents the relative magnitude of ‘effort’ and reward for intervening in each of the four zones (see Fig. 2), shown on the vertical axis in increasing magnitudes of ‘leverage’, from smallest to largest: Parameter, Process, Pathway, Purpose. The first ‘P’, prompts the actor to situate the Problem at the right level, and then the remaining four ‘P’ describe the places to intervene in the system.

The Five Ps provides a simple scaffold to allow actors explore the framing of a perceived problem, and encourages the exploration of different exploratory hypotheses through responses and interventions that effect change over short- and long-term timeframes. As a scaffold for exploring different system behavior, we propose that the simple act of hypothesis generation using the Five Ps allows practitioners to engage with systems thinking principles, such as examining the relationships between the parts and the whole, without the need for formal training in methodologies. To illustrate the domains and zones within the Five Ps, we will describe each briefly in relation to Responsible AI.

Problems identified in the *Parameter* zone are tractable (modifiable, mechanistic) characteristics of an AI system that are commonly targeted to improve the responsibility of AI. Examples include smaller visible flaws that are usually addressed through engineering solutions such as tweaking algorithms and parameters. The effort to fix these is relatively small, and changes in this zone are incremental and may have a negligible effect on the problem’s underlying structure or dynamics. They are important markers of the problem, but they are often symptomatic and not the root cause of the problem.

Problems identified in the *Process* zone consider the wide range of interactions between the feedback elements of an AI system that drive the internal dynamics, including social and technical processes associated with how the AI is designed, built, and deployed. This might include activities that speed up development times, or actively responding to emerging trends in the data. Changes in this zone are likely to result in resolving issues as they emerge or amplifying the positive and negative effect of assumptions.

Problems identified in the *Pathway zone* consider the ways through which information flows, the rules are set, and the power is organized. For example, improving transparency of how algorithms are employed, the governance or legislation of their use, or putting the ownership of data back into the consumer’s hands. These changes are structural to the system that allows the AI to operate, and result in establishing new patterns of behavior and agency.

Issues identified in the *Purpose* zone have the most potential to affect change in a system. These relate to the norms, values, goals, and worldviews of AI developers that are embodied in the system. It includes the underpinning paradigms based on which the system is imagined, and the ability to transform entirely and imagine new paradigms. Framing perceived problems in this zone serves to act as a compass to guide the developers to align with the fundamental purpose of the system.

The Five Ps—*problem, parameter, process, pathway, and purpose*—characterize five ways we can improve systems thinking literacy in relation to conceptualize changing the current state of Responsible AI towards the desired state of Responsible AI. Zones within the Five Ps are interrelated, and scale and reach also plays a role in the extent to which the system’s behavior changes. We propose that Five Ps are not part of a fixed hierarchy of change but serve as a conceptual tool to categorize and coordinate strategies to effect change towards improvement in Responsible AI.

## The Five Ps as an analytical tool

Reviewing the ongoing attempts to address Responsible AI, it should not surprise us that different problem framing leads to different responses. This is observed in the literature: when Responsible AI is a problem of technical and design flaws, it requires engineering fixes or a better design process (Lu et al., 2022; Soklaski et al., 2022), or leads to the development of tools, such as those that improve model explainability (Arrieta et al., 2020) and reducing biases (Sen & Ganguly, 2020); when the problem space expands to challenging questions about the underlying assumptions, visions, and the foundational purpose of the system, Responsible AI is understood as the microcosm of cultural and political challenges faced in society (Coeckelbergh, 2022; Mittelstadt, 2019), beyond technical and design issues.

Meadows (1999 p. 7) describes this framing problem through the Sufi story of the blind men and the elephant, where each blind man draws incomplete conclusions about the nature of an elephant by examining its parts rather than its whole. As an analytical tool, the Five Ps can help the actor/intervener to consider how a problem is framed and how this might interact with other efforts to address the same problem. To illustrate how the Five Ps can be applied as an analytical tool, consider an AI system that is used in a social media company causing misinformation and extremism.

In the Parameters zone, a typical response may include directly tweaking algorithms to analyze and address the biases to improve model outputs (i.e. the Reaction strategy in Fig. 2). In the longer term, this may lead to the development of software tools to translate principles of responsible AI, such as fairness, explainability, and accountability to improve the models. By taking these measures, the company should expect to control misinformation in its content-moderation models across the platform, which potentially leads to an improved user experience.

These efforts for quantifying, computing or mathematizing responsibility could be described as ‘technological solutionism’, built on a premise that the challenge of responsibility is a challenge of fixing a design flaw in the algorithms (Green, 2021; Häußermann & Lütge, 2021; Mittelstadt, 2019; Powles & Nissenbaum, 2018). In our example, although visible content moderation could improve, the paradigm under which the platform operates remains unchanged. If the company’s business model does not take into consideration other zones, engaging changes that undermine the company’s paradigms are unlikely to be supported. For example, a for-profit company is unlikely to support initiatives that have potential to reduce revenue streams (Hao, 2021; Lauer, 2021).

Typical responses in the Process zone may include intentionally promoting diversity and inclusion in development teams, publishing new professional guidelines and promoting training opportunities. As more diverse views are involved in the development of the model, assumptions are questioned and resolved during the development cycle. An intervention at this level has potential to adjust and adapt practices to changes in the operating environment.

Further, responses in the Pathway zone could include initiating governance structures within their firm for Responsible AI, such as review boards and roles and responsibilities for assuring that AI products and processes are ethical and aligned with AI principles the company abides by. Collective partnerships can also focus discussion on the development of design principles, guidelines, and best practices for AI (Jobin et al., 2019).

However, a unified and strong regulation does not yet exist, which can establish fiduciary duties to the public, and that implies the societies can just hope that reputational risks or company’s own values and standards may create more responsible approaches towards AI development and use (Kish-Gephart et al., 2010). Partnerships thus far have produced “vague, high-level principles and value statements, which promise to be action-guiding, but in practice provide few specific recommendations and fail to address fundamental normative and political tensions embedded in key concepts for example, in fairness and privacy” (Mittelstadt, 2019, p. 1).

Finally, in the Purpose zone, the same company could deploy resources to reconsider or redefining the purpose of their system, such as a fundamental change in purpose from ‘maximizing engagement’ to activities such as ‘truth-seeking’ or ‘social cohesion’. There are, for example, several experimental products, such as a platform called Polis, that highlight diverse views and work towards maximizing ‘consensus’ rather than ‘engagement’, and thereby fundamentally changing the goal of the system. In other words, by problematizing the problem in the Purpose zone, we are able to ask: “AI solution is the answer to what problem and why we want it?” (for the same discussion about ChatGPT, see Nabavi, 2023).

This simple example demonstrates that there are often multiple interactions between leverage zones, which can be studied for consideration of the intervention’s ‘effectiveness’, that is doing the right thing rather than doing things right. A systems thinking view prompts actors to consider the whole picture, and recognize that these zones are not discrete, and for effective implementation of change there should be consideration of the interactions required in combination across an entire system need to be aligned to realize change.

As an analytic tool, the Five Ps can be used to view the relative strength of interventions towards Responsible AI. In the following section, we look at how the Five Ps can also be used as a planning tool by those seeking to deliver Responsible AI.

## The Five Ps as a planning tool

A systems thinking view can help to address the ‘deeper’ questions about the governing rules, structure, business model, and purpose of a system. To move towards Responsible AI, we argue that interventions should be seen and studied in a holistic manner, not in isolation, to avoid missing linkages between the leverage zones, to prioritize competing efforts, to consider the narrow and broad consequences, and to plan in the short and long term.

As a planning tool, the Five Ps can be used to prompt consideration of the causal effects of solution to a given problem at multiple levels to achieve the desired level of ‘response’: *if we do this, then that will happen*. In Table 2, we provide a set of questions for each leverage zone that could be considered when considering a potential intervention. These questions should be seen as a general set of considerations: they are not exhaustive, and should be tailored to the situation at hand. By proactively considering questions that address systems-level concerns within each of the leverage zones, the problem can be properly assessed, and possible synergies and contradictions that might arise can be considered.

**Table 2:** Lines of questioning on interventions for Responsible AI.

Parameter questions	Process questions	Pathways questions	Purpose questions
How to keep the system stable with minimum change?	How should principles be drawn up and applied?	How can we change the structure of the system?	Why are we doing it? What are the goals?
How to step out of ‘abstract’ discussion by defining ‘practical’ actions?	How can we speed up things that are working?	What are the rules and who makes them (incentive, punishment, constraints)?	What are the fundamental assumptions behind our work? Do we need to change them?
How should principles be quantified?	How can we slow down things that are not working?	Who does and does not have access to what kinds of information?	How does our value system shape our work and the final product?
How risks and benefits can be managed through changing parameters and resources?	How can we reduce delays?	How can we share information more readily?	How our priorities drive the design choices we make?
What parameters need to be measured and modified?		How can we involve users in problem solving?	Are our motivations transparent and for the public good?
What resources can be deployed?		How do we know we are right? and, what is involved if things go wrong?	Who will benefit, who will lose?
What other impacts can we anticipate?			Are there other alternatives? What are they?

By exploring these questions, the Five Ps approach first allows decision-makers to better position and align interventions to the change they are seeking, and specifically avoid engaging with the system in siloed leverage zones, such as focusing on AI Principles alone or developing tools and practices for explainable models (see examples in Table 1). It recognizes and promotes the importance of ‘question-asking’ and how it can influence the shape of the pathway towards Responsible AI.

Second, it shows how focusing interventions within discrete leverage zones can precipitate through feedback processes in others, across various depths. The interdependencies between different leverage zones are important to be recognized and studied. Working from the deeper leverage zones shapes and limits the types of interventions available in shallower leverage zones (see Abson et al., 2017).

Third, it provides an aid for maintaining a holistic view over the challenges associated with Responsible AI, avoiding ‘atomized’ and ‘siloed’ conceptualizations in which social, technical, and governance aspects of AI systems are addressed separately (as constructed in Table 1), rather than elements that are tightly interacting togethers. The alternative is that we will remain in the existing paradigm, which mostly overlooks the structures, norms, values, and goals underpinning the complex problems Responsible AI is facing at deeper levels. Nevertheless, given the scale of existing social and ethical problems that have emerged in relation to the AI use, there is a strong incentive for major AI companies to adopt new tools and frameworks in order to prevent the development technologies that have the possibility to cause harm (McLennan et al., 2020).

And lastly, it provides a transdisciplinary context for a conversation about Responsible AI. Since AI developers come from varied disciplines (each with their own epistemic culture and ethical standards), to speak about Responsible AI, we need frameworks that can engage all stakeholders in meaningful discussions. This is particularly important as we can expect that experts interested in human and environmental aspects of AI-powered technologies are increasingly joining the conversation (Guzman & Lewis, 2020; Nabavi et al., 2019). The Five Ps framework provides a new communication tool for a wide range of stakeholders to speak about their ideas and priorities for the future of AI and collaborate using qualitative and quantitative methods.

## Conclusions and implications

Responsible AI needs to engage with the deep questions to find solutions that can address root causes that have led to negative outcomes in AI products and processes. As such we need to constantly reflect about whether the planned initiatives can realize the system shift required to create an environment conducive towards Responsible AI. To this end, we propose that the Five Ps framework is a useful tool to improve systems thinking literacy and to frame a conversation around alignment of initiatives to move existing systems towards a better representation of Responsible AI.

As an analytical tool, the Five Ps can help to make sense of the success of combinations of interventions. However, further work is required to study the short- and long-term effects of decisions arising from employing the Five Ps zones as a planning tool in practice. As an analytical tool and planning tool, we anticipate that conceptually simple frameworks that consider interventions through a systems thinking lens, such as the Five Ps, will yield better results over taking a fragmented, siloed approach.

The application of systems thinking principles in the field of Responsible AI is in its infancy, and the Five Ps represents one of dozens of systems thinking approaches that could be employed to move towards Responsible AI. To explore the capabilities, challenges and advantages of systems thinking tools such as the Five Ps, further work and development of real-world case studies that evidence the technique as a planning and evaluation tool is required.

A number of considerations for systems thinking in relation to Responsible AI remain open in relation to the identified challenges of taking a holistic view and working across disciplines. Parameters zone questions prompt us to consider how to coordinate and measure parameters that have an impact on Responsible AI, such as fairness, bias and accountability, across system and disciplinary boundaries. Process zone questions prompt us to consider how to enable efforts that enhance Responsible AI principles and mitigate unintended consequences, encouraging coordination of the effectiveness and efficiency of initiatives. A major consideration in the pathway zone is the notion of accountability and ownership of responsibility, including ensuring that structures evolve collaboratively with respect to advances in technology and changing societal expectations. Purpose zone questions prompt us to consider how to align multiple stakeholders with multiple perspectives to common goals in relation to Responsible AI, and how these goals interact and evolve with other paradigms that emerge.

This paper demonstrates that techniques from systems thinking can inform us on the pathway to Responsible AI. The Five Ps framework is a simple tool for systems thinking, allowing those working towards Responsible AI to develop a shared understanding of the likely long-term effectiveness of proposed initiatives; identify interdependencies between initiatives required for long-lasting change; provide frames of question-asking when considering initiatives; removal of barriers around silos of activity; consideration of the broader implications of initiatives, and; enable a transdisciplinary context for the conversation.

## Data Availability

Data sharing is not applicable to this research as no data were generated or analyzed.
